# Liquid biopsy guides successful molecular targeted therapy of an inoperable pediatric brainstem neoplasm

**DOI:** 10.1038/s41698-024-00535-8

**Published:** 2024-02-22

**Authors:** Cecilia Arthur, Lena-Maria Carlson, Jan Svoboda, Ulrika Sandvik, Cecilia Jylhä, Magnus Nordenskjöld, Stefan Holm, Emma Tham

**Affiliations:** 1 Clinical Genetics, Karolinska University Hospital, 171 76 Stockholm, Sweden; 2 Department of Molecular Medicine and Surgery, Karolinska Institutet, 171 76 Stockholm, Sweden; 3 Pediatric Oncology, Karolinska University Hospital, 171 77 Stockholm, Sweden; 4 Department of Women’s and Children’s Health, Karolinska Institutet, 171 77 Stockholm, Sweden; 5 Department of Pediatric Radiology, Karolinska University Hospital, 171 76 Stockholm, Sweden; 6 Department of Clinical Neuroscience, Division of Neurosurgery, Karolinska Institutet, 171 77 Stockholm, Sweden

**Keywords:** Predictive markers, Molecular medicine, CNS cancer, Paediatric cancer

## Abstract

Midline CNS tumors are occasionally inaccessible for surgical biopsies. In these instances, cell-free DNA (cfDNA) may serve as a viable alternative for molecular analysis and identification of targetable mutations. Here, we report a young child with an inoperable brainstem tumor in whom a stereotactic biopsy was deemed unsafe. The tumor progressed on steroids and after radiotherapy the patient developed hydrocephalus and received a ventriculoperitoneal shunt. Droplet digital PCR analysis of cfDNA from an intraoperative cerebrospinal fluid liquid biopsy revealed a *BRAF* V600 mutation enabling targeted treatment with MEK and BRAF inhibitors. The patient, now on trametinib and dabrafenib for 1 year, has had substantial tumor volume regression and reduction of contrast enhancement on MRIs and is making remarkable clinical progress. This case highlights that in a subset of CNS tumors, access to liquid biopsy analysis may be crucial to identify actionable therapeutic targets that would otherwise go undiscovered.

## Introduction

Pediatric low-grade gliomas (pLGG) are the most common central nervous system tumors in children^1^. Initial management relies on complete surgical resection, whenever feasible, and histopathological examination. Approximately half of patients will need adjuvant therapy after initial surgery. Historically, this has been achieved by adjuvant chemotherapy or, less frequently, radiotherapy^2^. Although the 10-year overall survival is excellent for pLGG in most tumor locations, thalamic and brainstem tumors have significantly lower survival rates, and upfront radiotherapy is associated with an increased risk of tumor-related death as well as increased delayed mortality^3^.

Improved molecular diagnostics have shown frequent somatic driver alterations affecting the MAPK pathway in pLGG, such as the *BRAF* V600E mutation or the *KIAA1549*::*BRAF* fusion^4–6^. *BRAF* V600-mutant pLGGs tend to respond poorly to standard-of-care chemotherapy and have worse progression-free and overall survival rates compared to patients with wild type (wt) or *BRAF*-rearranged tumors^7–9^. However, treatment of *BRAF* V600-mutant pLGG with MEK-inhibitor trametinib, with or without BRAF-inhibitor dabrafenib, has shown clinical efficacy and tolerability in this patient group with response-rates exceeding those of conventional chemotherapy^10,11^. Recent studies on pLGG show that efficacy appears to be greater with the combination therapy regimen, and that such treatment offers significantly longer progression-free survival than chemotherapy^12,13^.

Last year, the trametinib and dabrafenib combination received Food and Drug Administration (FDA) approval for treatment of pediatric and adult solid tumors, including pLGG, after having shown pan-cancer activity across 21 histologies^14–17^. In order to initiate treatment, however, a molecular diagnosis must be made. Brainstem surgery has a considerable rate of intraoperative and postoperative complications, and while stereotactic biopsies are commonly safe procedures, occasionally, neither surgery nor safe surgical biopsies can be performed^18–21^. In these scenarios, a liquid biopsy may serve as a minimally invasive alternative to identify molecular therapeutic targets^22,23^.

## Results

### Case presentation

A previously healthy, 3 year and 10-month-old girl, born in gestational week 34, presented at the emergency department with a 6-week long history of balance disturbance, squinting, dysphagia, difficulty passing urine, and left-sided torticollis. The parents were previously healthy with no family history of heritable disorders. An initial CT scan, followed by a brain MRI with spectroscopy, verified a tumor measuring 5.5 × 3 × 2.5 cm located in medulla oblongata with exophytic growth into the 4th ventricle, involvement of inferior pons and left middle cerebellar peduncle, and caudal extension into cervical medulla with surrounding edema. The radiologic characteristics suggested a low-grade brainstem glioma. The tumor was considered inoperable and a safe surgical biopsy could not be performed. The patient was initiated on steroids with good clinical response. A lumbar puncture for liquid biopsy analysis was discussed with the caregivers but they declined the procedure at this point. To obtain local control, a low-grade glioma (LGG)-compatible chemotherapy regimen with vincristine and carboplatin, or irradiation, was suggested to the caregivers. However, they opted for a watch-and-wait strategy.

Two and a half months after initial diagnosis, the child was admitted due to hypoventilation when undergoing anesthesia for an MRI, suggesting an impaired central respiratory drive. The MRI showed progress in tumor size and the dose of steroids was increased to reduce symptoms. A subsequent MRI 1 month later revealed further tumor growth and signs of incipient hydrocephalus. Photon therapy was commenced 4 months after diagnosis, with a treatment plan for 1.8 Gy × 30 under the support of an increased daily dose of steroids.

**Fig. 1:**
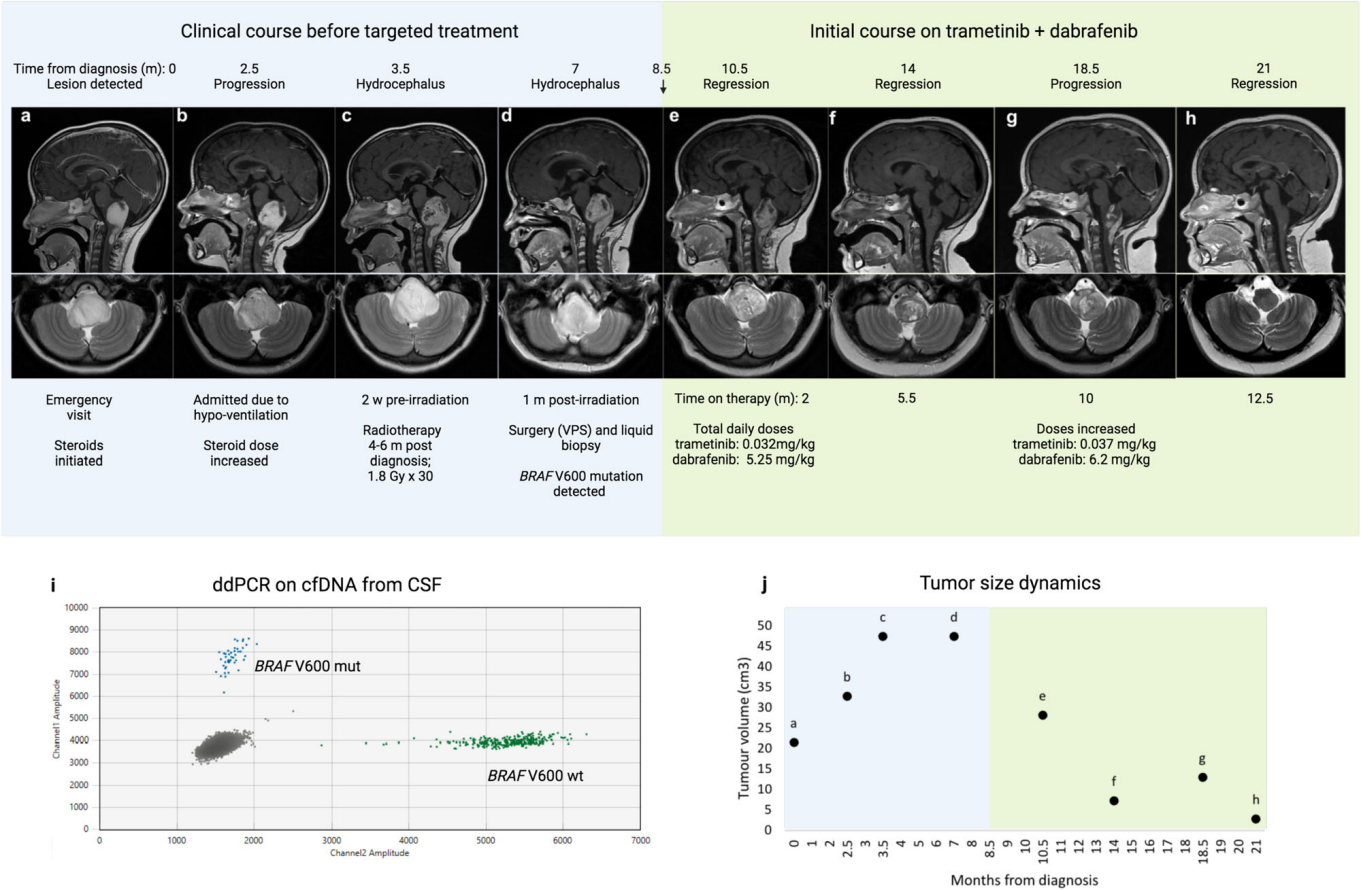
Response to trametinib and dabrafenib. Clinical course of the patient (**a**–**h**) shown as T1-weighted sagittal MRI images with contrast (top panel) and T2-weighted axial scans (bottom panel). Arrow denotes start of targeted therapy. CSF liquid biopsy results (**i**) as a 2D fluorescence amplitude plot. *Y*-axis: end-point FAM-fluorescence from target (*BRAF* V600E/R/K). *X*-axis: end-point HEX-fluorescence from wt sequence. Changes in tumor volume over time (**j**) based on MRIs using ellipsoid volume estimation (4/3*π*x*y*z/8 with x, y and z representing tumor diameters in cm). Background colors represent pre (blue) and post (green) targeted treatment periods. M months, w weeks, ddPCR droplet digital PCR, cfDNA cell-free DNA, CSF cerebrospinal fluid. Created with BioRender.com.

After eight given fractions of irradiation, the patient acquired a bilateral pneumonia and a need for treatment with broad spectrum antibiotics and BiPAP ventilation due to low respiratory drive and carbon dioxide retention. Radiotherapy could recommence after three and a half weeks of disruption, and all 22 remaining fractions were given continuously, in total 54 Gy, finishing ~2 months after initiation of irradiation. Major clinical events, therapeutic interventions and outcomes are shown as a timeline in Fig. 1a–h.

Less than 1 month after completion of radiotherapy, a papilledema was detected during ophthalmological examination and MRI confirmed increased hydrocephalus. Ventriculo-peritoneal shunting (VPS) was performed shortly thereafter. Cerebrospinal fluid (CSF) and blood samples (2 and 4 ml, respectively) were collected during VPS in Cell-Free DNA BCT tubes (STRECK, La Vista, NE, USA). Cell removal by double centrifugation was performed on the same day. For further details on processing of samples, see “Methods”. Droplet digital PCR (ddPCR) using a commercial *BRAF* V600 screening kit revealed that CSF was clearly positive for circulating tumor DNA (ctDNA) with 45 mutant and 284 wt copies, corresponding to a variant allele frequency of 13.6% (Fig. 1i). Plasma was ctDNA negative with 952 wt copies.

Results were reported back to the treating physicians within 5 days from sampling. CSF that had been collected during the VPS procedure (5 ml) also sent for cytological analysis and showed no malignant cells.

Despite VPS, the patient’s general condition worsened slightly with increased vertigo over the weeks to come, indicating that the patient was in need of further treatment. Upon confirmation of a *BRAF* V600 mutation in CSF, a license was sought from the Swedish Medicines Agency to give targeted therapy^24^. Once approved, treatment was initiated 8.5 months post diagnosis with dabrafenib (total daily dose 5.25 mg/kg, divided into two daily doses) and trametinib (starting dose 0.025 mg/kg/day, increased to 0.032 mg/kg/day after 2 months)^12,25^.

The patient’s overall neurological function gradually improved after initiation of targeted therapy. After 2 weeks, her overall eye status had improved and after 2 months, she was able to run and utilize a tricycle. Her eye movements improved further according to the parents’ description, and her ability to swallow stabilized. She had one episode with suspected seizures, which were verified by electroencephalogram. Anti-epileptic treatment with levetiracetam was initiated (150 mg gradually increased to 550 mg, twice daily) with subsequent cessation of symptoms.

Two and five and a half months into treatment, two MRIs had shown reduction of contrast enhancement and substantial tumor volume regression (in total 40 cm^3^). Around the same timepoints, sleep studies were performed that showed desaturations motivating BiPAP treatment at night. Nine months after the start of targeted therapy the patient was making advances in her clinical progress with further improvements in speech, eye movements, swallowing, balance, strength in extremities and gross motor skills, now being able to jump on one leg and ride a bicycle. Due to a grade 3 weight gain (according to the National Cancer Institute Common Terminology Criteria for Adverse Events v. 5.0) the patient was referred to a nutritionist^26^. Nonetheless, the weight gain continued. A subsequent MRI, performed 10 months into treatment, showed an increase in tumor size and a PET scan confirmed presence of viable tumor tissue. At this point, doses were increased stepwise; dabrafenib to a total daily dose of 6.2 mg/kg and trametinib to 0.037 mg/kg. The next MRI, ~2 months after treatment intensification, again showed tumor regression. Tumor size dynamics are shown in Fig. 1j. The child, now on targeted treatment for 1 year, has no neurological symptoms but still needs a BiPAP ventilator at night.

## Discussion

This case report illustrates that a tumor-agnostic liquid biopsy may successfully guide clinical precision treatment of a pediatric CNS neoplasm that is inaccessible for surgical biopsy. Although the minimalistic approach used here, with ddPCR for a single nucleotide variant, does not provide a comprehensive molecular diagnosis, it has aided clinical decision-making and enabled targeted molecular treatment of a tumor that would, most likely, have responded poorly to standard-of-care chemotherapy^8,27^. *BRAF* V600 analysis by ddPCR is a clinically validated method at our laboratory (accredited by Sweden’s national accreditation body, SWEDAC, and validated through the European Consortium for Histiocytosis, ECHO)^28,29^.

Detection of *BRAF* V600 mutations in different kinds of liquid biopsies from children with CNS tumors has been reported earlier, however, these reports describe either tumor-informed detection, or tumor-agnostic detection without subsequent targeted treatment^30–34^. The same holds true for reports on adult cancer patients in which CSF was analyzed for *BRAF* mutations^35–37^. We have found one pediatric case (of diffuse leptomeningeal glioneuronal tumor) in which CSF-analysis contributed to targeted treatment. In this case, a surgical biopsy was performed but failed to provide molecular information, and a *KIAA1549*::*BRAF* fusion was detected in cell-free DNA (cfDNA) using a next-generation sequencing panel. Treatment with a MEK inhibitor was initiated, however, this was 3.5 years after initial presentation of symptoms and 1.5 years after the surgical biopsy. The patient is described as stable 3 months into treatment. The overall findings of the report, of 45 pediatric, adolescent and young adult patients with CNS tumors, and liquid biopsy analysis using a next-generation sequencing assay, support broader implementation of clinical cfDNA CSF testing to improve the care of patients in these age groups^22^. However, the clinical utility may be limited to identifying, rather than ruling out, actionable alterations as there is a multitude of biological and technical confounders that can cause false negative results (including tumor location and size, low rates of apoptosis, small sample volumes and other subsampling errors)^22,38^. The false negative rates, however, may be lower than those of CSF cytology^39,40^.

While liquid biopsies hold promise for many purposes in glioma management (establishment of a diagnosis when tissue is unavailable, monitoring of residual disease, detection of early relapse, distinguishing progression from pseudo progression by imaging and prediction of outcome) standardized pre-analytics and large clinical trials are needed to validate findings^23,41–46^. The European Society for Paediatric Oncology Brain Tumor Group (SIOPE BTG) Liquid Biopsy Consensus Initiative strives to contribute to such standardization and validation within Europe^47^.

We would like to emphasize that in the case presented here, there was no extra puncture for the purpose of drawing a liquid biopsy but rather we made use of biofluids that would normally be shunted to the peritoneal space. As many children with CNS tumors require CSF diversion^48^, we would like to encourage others to biobank residual CSF samples as they are possible to analyze retrospectively. If substantial volume is available this may allow for targeted or broad analysis of cfDNA or of other liquid biopsy components (circulating tumor cells, extracellular vesicles, micro-RNAs, proteins, etc.)^49–51^. Massive parallel sequencing approaches, such as low coverage whole genome sequencing or gene panel sequencing, and global methylation profiling techniques may be applied to overcome the limitation of ddPCR in interrogating only a small number of mutations.

The patient that we describe continues to have clinical response to combination treatment with dabrafenib and trametinib 1 year after initiation. However, as the tumor was not treatment naïve, and transient pseudo progression is not uncommon after irradiation of pediatric low-grade glioma, we cannot exclude that radiotherapy contributed partially to the initial response. The plan is to continue targeted treatment until disease progression, or unacceptable toxicity. While targeted treatment may confer fewer side and late effects of treatment than chemo- or radiotherapy, a majority of patients who receive trametinib/dabrafenib treatment do experience some type of adverse event. However, adverse event-related treatment discontinuations in children are more common with trametinib monotherapy than with trametinib plus dabrafenib treatment^10,12,16,52^. As for the observed side effects in this case, no endocrinological deficit seems to underlie the weight gain. While continuous steroid treatment during the first year after diagnosis and lifestyle factors may have contributed to the weight increase, weight gain has been observed in 44% of children with pLGG on the same combination treatment in a recent trial^13^.

In conclusion, ultra-sensitive diagnostic liquid biopsy analysis on surplus biofluids for selected molecular targets was clinically useful to direct precision treatment of an inoperable brainstem tumor in a small child with limited therapeutic options and negative CSF cytology.

## Methods

### cfDNA isolation

Liquid biopsies were collected in Cell-Free DNA (cfDNA) BCT tubes (10 ml) (STRECK, La Vista, NE, USA). The entire sample volumes, 2 ml of CSF and 4 ml of blood, underwent cell removal by double centrifugation (10 min at 4 °C 1600 × *g* and 16,000 × *g*) on the day of collection. Supernatants, 2 ml of CSF and 2 ml of plasma, were frozen at −80 °C over the weekend. Cell-free supernatants were then thawed and cfDNA was isolated using the QiAamp Circulating Nucleic Acid Kit on the QIAvac24 Plus vacuum manifold (Qiagen, Manchester, UK). cfDNA was eluted in 40 μl of AVE buffer and stored at RT. Plasma from blood donors was processed in the same manner and used as negative control (NC).

### ddPCR

Triplicate ddPCR reactions on non-amplified cfDNA from CSF and plasma were run on the QX200 AutoDG Droplet Digital PCR System/QX200 Droplet Reader (BioRad, Hercules, CA, USA) according to the manufacturer’s instructions using the *BRAF* V600E/K/R screening kit (cat #12001037, BioRad, Hercules, CA, USA. MIQE Context: hg19|chr7:140453075-140453197). Reactions contained 10 μl 2x ddPCR Supermix for probes (No dUTP) (BioRad, Hercules, CA, USA), 1 μl 20x *BRAF V600* Screening Assay and 11 μl of cfDNA eluate. Triplicates of no template control (nuclease free water) and positive control samples, and 15 wells of NC cfDNA from donor plasma, were run on the same plate. Thermal cycling conditions were: 1 cycle at 95 °C for 10 min, 40 cycles at 94 °C for 30 s and 55 °C for 1 min, 1 cycle at 98 °C for 10 min, and 1 cycle at 4 °C ∞ at ramp rates of 2 °C/s. Results were visually reviewed applying Poisson and total error models with a 95% confidence interval using the QX Manager^TM^ Software Standard Edition Version 1.2 (BioRad, Hercules, CA, USA).

The report complies with all relevant ethical regulations stated by the Swedish Ethical Review Authority, including the Declaration of Helsinki. Written informed consent has been obtained from the patients’ guardians/next of kin to participate and to publish this paper.

### Reporting summary

Further information on research design is available in the Nature Research Reporting Summary linked to this article.

### Supplementary information

Supplementary Table 1

REPORTING SUMMARY

## Data Availability

The output data table from QX Manager^TM^ Software Standard Edition Version 1.2 (BioRad, Hercules, CA, USA) is available as Supplementary Table 1. Raw data in the form of qlp/ddPCR-files are available from the corresponding author on reasonable request.
